# Risk perception of automotive fuel poisoning among gas station attendants

**DOI:** 10.47626/1679-4435-2022-745

**Published:** 2023-02-03

**Authors:** Larissa Guerino Ferla, Gustavo Henrique Oliveira da-Rocha, Rômulo Tadeu Dias de-Oliveira, Éric Diego Barioni

**Affiliations:** 1 Biomedical Sciences Undergraduate Program, Universidade de Sorocaba, Sorocaba, SP, Brazil; 2 Department of Clinical and Toxicological Analyses, Universidade de São Paulo, São Paulo, SP, Brazil

**Keywords:** benzene, occupational hazards, toxicology, gas station, benzeno, riscos ocupacionais, toxicologia, posto de combustível

## Abstract

**Introduction:**

Everyday, gas station attendants ate exposed to numerous toxic substances found in fuels. Benzene stands out among these toxic chemical agents; depending on its concentration, it can cause mucosal irritation or even pulmonary edema. A considerable number of gas station attendants is aware of the risks associated with benzene poisoning, but they are not aware of the risks associated with other automotive pollutants.

**Objectives:**

To evaluate and understand the risk perception of automotive fuel poisoning among gas station attendants in the Sorocaba region, state of São Paulo.

**Methods:**

Sixty gas station attendants were evaluated in the Sorocaba region. Data were collected between October 2019 and September 2020 using a semi-structured, individual, closed-ended questionnaire whose questions identified the participants’ perception and aimed to analyze: the general profile of the studied population; practices for handling fuels and knowledge on their toxic effects, use and instructions of personal protective equipment, symptoms possibly associated with fuel exposure, the participants’ perception of poisoning risks, and their participation in occupational medicine programs.

**Results:**

The obtained results demonstrated that most gas station attendants wore at least basic personal protective equipment, and some of them reported symptoms linked with benzene exposure. Still, a considerable number of employers does not provide adequate training to gas station attendants, which is possibly associated with inadequate use of personal protective equipment.

**Conclusions:**

Our data showed indications of non-compliance by gas station attendants as to the use of personal protective equipment at the workplace, and by employers as to the provision of adequate training.

## Abstract (pt)

**Introdução:**

Todos os dias, trabalhadores frentistas são expostos a inúmeras substâncias tóxicas presentes nos combustíveis. Entre os agentes químicos tóxicos, destaca-se o benzeno que, dependendo da concentração, pode causar irritabilidade de mucosas e até edema pulmonar. Um número considerável de frentistas conhece os riscos associados à intoxicação por benzeno, porém desconhece os riscos causados pelos demais poluentes automotivos.

**Objetivos:**

Avaliar e compreender a percepção de risco de intoxicações por combustíveis automotivos em frentistas da região de Sorocaba, São Paulo.

**Métodos:**

Foram 60 frentistas avaliados na região de Sorocaba. A coleta de dados foi realizada de outubro de 2019 a setembro de 2020, a partir de um questionário semiestruturado, individual e fechado, cujas questões identificaram a percepção dos sujeitos envolvidos, buscando analisar: perfil geral da população estudada; práticas que envolvem o manuseio de combustíveis e o conhecimento sobre efeitos tóxicos; uso e instruções quanto aos equipamentos de proteção individual; sintomas possivelmente associados com exposição a combustíveis; percepção dos sujeitos quanto ao risco de intoxicações; participação em programas de medicina ocupacional.

**Resultados:**

Os resultados obtidos demonstraram que boa parte dos frentistas utiliza ao menos equipamentos de proteção individual básicos, e que alguns reportam sintomas ligados à exposição ao benzeno. Ainda, um número considerável de empregadores não fornece treinamento adequado aos frentistas, o que possivelmente se associa com uso inadequado de equipamentos de proteção individual.

**Conclusões:**

Os dados apresentados mostram que existe indícios de inobservância por parte dos frentistas quanto ao uso de equipamentos de proteção individual no local de trabalho e por parte de empregadores quanto à disponibilização de treinamento adequado.

## INTRODUCTION

The profession of gas station attendant or pump attendant is described by number 5211-35 in the Brazilian Occupational Classification (Classificação Brasileira de Ocupações [CBO]).^1^ Gas station attendants work as salaried workers with formal contracts or as autonomous workers at commercial companies. Gas station attendants work in outdoor environments but are in contact with numerous volatile toxic substances such as automotive fuels. This profession requires lower secondary education only, and this minimum requirement can compromise risk perception regarding fuel handling procedures and their toxicity.^1^

Throughout the years, scientific literature has shown that gas station attendants accumulate risk behaviors during their job, such as the inappropriate use of personal protective equipment (PPE), topping off the gas tank and pumping gas close to the face, and insufficient risk perception and guidance by the establishment. Additionally, studies aiming to demonstrate the perception, by gas station attendants, of the risk of automotive fuel poisoning and the employers’ attitudes (such as guidance and provision of PPE and medical support to workers) are scarce, and this lack of information can contribute to an increased number of poisoning cases and the absence of public policies that favor the general health and wellbeing of workers. During their job, gas station attendants are subject to various health hazards and health problems, and the exposure to fuels and chemical products are the most harmful to the health-disease process.^2^

Benzene stands out among the chemical agents present in different types of fuels. It is a flammable liquid with a pleasant smell, but toxic even at low concentrations; benzene can be absorbed by the body through the skin or by breathing. One of the toxic effects of its absorption is central nervous system depression, including symptoms such as sleepiness, tremors, dizziness, and headaches.^3^ Apart from the effects to the central nervous system, myalgia is a common toxic effect of benzene exposure.^4^ Other toxic substances in addition to benzene can be found at gas stations, such as ethanol, gasoline, and diesel fuel. According to Lopes,^5^ when in contact with the eyes, ethanol can cause eye injuries; when in contact with the skin, it can cause skin irritation; if inhaled, it can cause headaches, loss of motor coordination, and loss of conscience. According to Moreira et al.,^6^ gasoline is basically a mixture of various substances, such as sulfur, oxygen, nitrogen, and metals. The most relevant symptoms of exposure to gasoline and its constituents are skin irritation, causing redness and dryness, and eye irritation, leading to redness, pain, and watery eyes.^7^ Moreover, gasoline exposure can cause respiratory tract irritation with coughing, sneezing, and shortness of breath; if ingested, it can cause sleepiness, vertigo, headache, nausea, and vomiting.^8^

Therefore, this study aimed to evaluate and understand the risk perception of automotive fuel poisoning among gas station attendants working in the Sorocaba region, state of São Paulo, between October 2019 and September 2020. We also aimed to determine how gas station attendants are instructed by companies, how they understand and experience poisoning, how they obtain information on the hazards of these products and the use of PPE, and how they react to dangerous situations.

## METHODS

A total of 60 gas station attendants in the Sorocaba region (including the municipalities of Sorocaba, Votorantim, and Porto Feliz), state of São Paulo, were selected for participating in this study by filling out a questionnaire on various aspects related to risk perception experienced in their work environment. All gas station attendants were informed of the questionnaire content and study objectives; all participants voluntarily contributed with the study. The questionnaire used in this study was semi-structured, individual, and closed-ended, being constructed by the authors (supplementary material). The items identified risk perception associated with the participants’ exposure, aiming to analyze data related to: practices when handling fuels; the use and supply of PPE and guidance on its use; information and guidance on product hazards; the participants’ perception of poisoning risks and cases of poisoning experienced by them; smoking habits and use of medications; working hours, and participation in occupational medicine programs. Male and female gas station attendants aged 18 years or older were interviewed, and a quantitative analysis of data was performed after tabulating the responses.

By grouping data contained in the forms into categories, we were able to structure a set of interconnected concepts that comprised the results presented in this study. The participants signed a free and informed consent form analyzed by the Research Ethics Committee of Universidade de Sorocaba (CEP/CONEP) and approved under No. 4387800, which authorized the use of their data in this study and assuming minimal risks of participating in it, such as the minimal exposure of personal data and unease when answering questions.

Researchers contacted the participants and visited their workplaces for performing the interviews.

In addition to the descriptive analyses of data compiled into categories, they were also analyzed via **χ**^2^ tests for verifying the association between categorical variables. Odds ratios and their respective p-values are shown. p-values < 0.05 were considered significant.

## RESULTS

Out of 60 gas station attendants interviewed in this study, 52 (86.7%) declared being male and 8 (13.3%) declared being female; 20 workers (33.3%) stated they were aged between 18 and 30 years, 12 (20%) said they were aged between 31 and 45 years, 14 (23.3%) were aged between 46 and 65 years, and 14 (23.3%) did not agree to inform their age. Regarding the time in this occupation, 9 gas station attendants (15%) declared they had this occupation for 1 year or less, 12 (20%) said they had this occupation for 1 year or more, and 39 (65%) declared having this occupation for 5 years or more. As to their education levels, 7 (11.7%) gas station attendants reported having lower secondary education, 47 (78.3%) had upper secondary education, 3 (5%) had higher education, and 3 (5%) did not agree to inform their education level. Additionally, 57 (95%) gas station attendants reported they worked 7 hours and 20 minutes per day, while 3 (5%) declared they had different working hours; 14 (23.3%) professionals reported they worked in 5/1 shifts and 43 (71.7%) worked in 6/1 shifts, that is, 5 or 6 working days for each day off. Three (5%) workers reported they had different working hours, and 12 (20%) out of 60 gas station attendants worked overtime. Finally, when inquired about their work shifts, 56 gas station attendants (93.3%) reported they worked during the day, whereas only 3 professionals stated they worked night shifts (5%), and 1 (1.7%) did not answer.

The absence of medical follow-up and/or training contributes to an increased risk of poisoning and health problems.^4^ In this sense, our study aimed to identify, through the gas station attendants’ answers, lifestyle habits related to disease development; we were able to notice that 1 worker (1.7%) reported alcohol consumption, 5 workers (8.3%) reported tobacco use, 20 (33.3%) reported coffee consumption, and 36 (60%) stated they had no addictions. Moreover, 21 gas station attendants (35%) reported regular physical activity and 13 (21.7%) reported they used long-term medications. The data described above are described in Table 1.

**Table 1:** Sociodemographic, lifestyle, and occupational data

Characteristics	n (%)
Gender	
Male	52 (86.7)
Female	8 (13.3)
Age group (years)	
18 to 30	20 (33.3)
31 to 45	12 (20.0)
46 to 65	14 (23.3)
Not informed	14 (23.3)
Time in occupation (years)	
1 or less	9 (15.0)
1 or more	12 (20.0)
5 or more	39 (65.0)
Education	
Lower secondary education	7 (11.7)
Upper secondary education	47 (78.3)
Higher education	3 (5.0)
Not informed	3 (5.0)
Daily working hours	
7h20min	57 (95.0)
Other type of workday	3 (5.0)
Weekly working hours	
5/1 shifts	14 (23.3)
6/1 shifts	43 (71.7)
Other type of workday	3 (5.0)
Work shift	
Day	56 (93.3)
Night	3 (5.0)
Not informed	1 (1.7)
Addictions	
Coffee	20 (33.3)
Smoking	5 (8.3)
Alcoholic beverages	1 (1.7)
No	36 (60.0)
Physical activity	
Yes	21 (35.0)
No	39 (65.0)
Disease	
Diabetes mellitus	2 (3.3)
Hypertension	4 (6.7)
Others	3 (5.0)
No	51 (85.0)
Use of medications	
Yes	13 (21.7)
No	47 (78.3)

We highlight the following PPE as necessary for performing the job: boots, pants and a shirt, protective hand cream, gloves, sunscreen, and protection goggles. The results demonstrated that 58 gas station attendants (96.7%) wore boots and pants, 57 (95%) wore a shirt, 19 (31.7%) used protective hand cream, 10 (16.7%) wore gloves and sunscreen, and 2 (3.3%) workers wore other pieces of equipment such as protection goggles.

The results obtained in this study demonstrate that companies have developed training programs up until a certain point. Even though 75% of the gas station attendants had been trained, a considerable fraction of these professionals (15 workers, 25%) had not been previously trained. Our study also aimed to identify whether the gas station attendants regularly underwent examinations and received medical support, and we verified once more a great variety in answers regarding the availability and frequency of medical visits. Out of all participants, 9 (15%) gas station attendants said that medical follow-up visits were provided by the company every 6 months; 2 (3.3%) stated that medical visits only happened at admission; 5 (8.3%) gas station attendants reported they had never been to a medical follow-up visit; 42 (70%) professionals said that medical follow-up visits were performed annually; and 2 (3.3%) were not able to answer. Regarding examinations for quantifying benzene and/or its metabolites in urine, our results demonstrated that more than half of the interviewees had never undergone a urine test at the company’s request. This way, whereas 20 (33.3%) gas station attendants reported they had been through urine tests, 40 (66.7%) professionals stated they had never undergone such tests. Table 2 illustrates the results regarding occupational data.

**Table 2:** Occupational data on gas station attendants participating in this study

Characteristics	n (%)
PPE	
Boots	58 (96.7)
Pants	58 (96.7)
Shirt	57 (95.0)
Protective hand cream	19 (31.7)
Gloves	10 (16.7)
Sunscreen	10 (16.7)
Protection goggles	2 (3.3)
Frequency of training sessions	
Monthly	6 (10.0)
Every 6 months	6 (10.0)
Annual	33 (55.0)
No training	15 (25.0)
Medical follow-up visits	
At admission	2 (3.3)
Every 6 months	9 (15.0)
Annual	42 (70.0)
Never	5 (8.3)
Not informed	2 (3.3)
Urine collection	
Yes	20 (33.3)
No	40 (66.7)

PPE = personal protective equipment.

Finally, the professionals answered questions regarding the signs and symptoms they had experienced during their period on the job. Most of the interviewed professionals reported they had never experienced any signs or symptoms. However, among those who reported having experienced symptoms, the number of gas station attendants reporting myalgia (9 workers, 20.7%) and allergic rhinitis (7 workers, 11.7%) is considerable. It is also worth noting that 4 (6.7%) gas station attendants reported vertigo, 5 (8.3%) reported headaches, and 6 gas station attendants (10%) reported feeling sleepy. Table 3 demonstrated the total number of gas station attendants who reported signs and symptoms.

**Table 3:** Signs and symptoms of poisoning associated with exposure to benzene or other fuels, as reported by gas station attendants

Symptoms	n (%)
Back pain	12 (20.0)
Myalgia	9 (15.0)
Allergic rhinitis	7 (11.7)
Sleepiness	6 (10.0)
Headache	5 (8.3)
Ocular hyperemia	4 (6.7)
Vertigo	4 (6.7)
Dyspnea	3 (5.0)
Xerostomia	3 (5.0)
Tachycardia	2 (3.3)
Tremors	2 (3.3)
Arrhythmia	1 (1.7)
No symptoms	39 (65.0)

The obtained data were categorized and correlated as demonstrated in Table 4, and a possible association of the use of PPE with time in occupation and the reported symptoms was verified. The results show no statistically significant association between the categorical variables analyzed in this study. The “closest” association to statistical significance was that between training and use of PPE, where gas station attendants who received some type of training were 3.2 times more likely to wear at least one piece of PPE apart from boots, pants, and a shirt; this result, although not significant, shows the importance of the training provided by employers, making gas station attendants more aware of the use of PPE and consequently reducing their exposure to toxic substances.

**Table 4:** Association of training, education, use of PPE, and time in occupation with symptoms associated with exposure to benzene and other toxic substances

Condition	p-value	Odds ratio
Training x use of PPE	0.1284	3.200
Education x use of PPE	> 0.9999	1.532
Headache x use of PPE	> 0.9999	1.079
Rhinitis x use of PPE	0.6971	0.6095
Myalgia x use of PPE	0.7215	1.347
Headache x time in occupation	> 0.9999	0.7917
Rhinitis x time in occupation	0.4037	3.636
Myalgia x time in occupation	0.4734	2.078

PPE = personal protective equipment.

## DISCUSSION

The use of PPE is essential for work as a gas station attendant, and it should be encouraged by employers. Similarly, employers should guarantee the availability of PPE for workers.

Although the studied population did not satisfactorily use the required PPE, we noticed a higher awareness when comparing our results to those of studies by Amaral et al.^9^ and Maciel et al.,^10^ where no gas station attendant reported using any type of PPE during the workday. However, Alves^11^ describes that the proper use of PPE is the only way to reduce the risks of exposure to toxic agents.

According to regulatory standard NR-06,^12^ which comprehends PPE, the company is obligated to provide functioning equipment in perfect state of conservation, in accordance with the hazards at the workplace. The company is responsible for guiding (and demanding from the employee) the use and conservation of PPE; the periodical cleaning and maintenance of PPE are also the company’s responsibility. However, no standards regulate and specify which PPE should be worn during exposure or which procedures and prevention measures should be taken when workers are exposed to benzene and other toxic substances present in fuels, which are frequently volatile.^2^ Still on the study performed by Rocha et al.,^2^ the authors observed that the main pieces of PPE worn by these professionals were boots and aprons, and few gas station attendants reported the use of other pieces of PPE such as pants, shirt, and chemical gloves. On the study performed by Cezar-Vaz et al.,^13^ on the other hand, gas station attendants reported that they wore gloves, shoes, and working clothes for minimizing risks.

Medical follow-up, which should be provided by the employer, aims to ensure the health of professionals and the quality of the work environment, identifying (or not) probable clinical and/or laboratory alterations that could demonstrate the exposure of a professional to chemical products beyond the permissible exposure limits. Laboratory examinations are able to not only demonstrate the presence of a substance or its metabolites, but also indicate organic dysfunctions by identifying cellular, enzymatic, or other alterations.^14^ Medical follow-up, as well as training sessions that should be provided by employers, should encourage the use of PPE by gas station attendants as well as healthy lifestyle habits, such as not smoking or drinking, regular physical activity, and healthy eating.

Even though companies carry out training sessions, a part of the professionals had never been at one, which highlights a lack of standardization of the frequency of these training sessions; these data are even more relevant when compared with the study by Cezar-Vaz et al.,^13^ where accidental fuel-skin contact and fuel inhalation occurred in 91.4% and 77.8% of the studied population, respectively.

When it comes to medical examinations, NR-07^15^ regulates the mandatory implementation of the Occupational Health Examination Program (Programa de Controle Médico de Saúde Ocupacional [PCMSO]), which aims to preserve the workers’ health and demands examinations at admission, periodically, when returning to work, when changing occupations, and at termination of employment. This standard also comprehends the frequencies with which periodical examinations should be performed^15^ and, according to article 58 of the Consolidation of Labor Laws (Consolidação das Leis do Trabalho [CLT]), the workday of a gas station attendant is predominantly a daytime shift of 6 to 8 hours. Moreover, Annex 13-A of NR-15^16^ classifies the gas station attendant occupation at of maximum hazard, since these professionals handle products containing toxic substances such as toluene, methylbenzene, and benzopyrene, among others. The biotransformation of benzene occurs primarily in the liver, and part of the benzene is eliminated in the urine via its biotransformed products (phenol, catechol, hydroquinone, phenylmercapturic acid, and trans,trans-muconic acid [tt-MA]). Urinary tt-MA detection is one of the most widely used techniques for determining benzene exposure,^17^ as described in Annex 1 of NR 07.^15^

As discussed above, we highlight benzene among the chemical agents to which gas station attendants are exposed, as it can cause mucosal irritation (mouth, nose, and eyes) and pulmonary edema via aspiration,^17^ in addition to other toxic effects such as central nervous system depression with periods of sleepiness, hypotension, headaches, nausea, tachycardia, dyspnea, and seizures.^4^ As previously mentioned, myalgia is described as common in cases of benzene exposure, corroborating a higher number of affected attendants, which suggests that workers in our study are somewhat exposed to benzene at their workplace.^4,18^

Nevertheless, the lack of other statistically significant associations does not necessarily indicate that the analyzed variables do not influence symptom manifestations; symptoms stemming from chronic exposure to low levels of toxic agents can appear at any point of an individual’s life and are associated with other predisposing, multifactorial conditions that are hardly reflected by a broad questionnaire such as the one used in this study. A larger sample size could also highlight associations that are obscured by the inherent variability of a small sample size. Other authors such as D’Alascio et al.^4^ reported that specific habits such as sniffing the gas cap to identify the fuel are associated with symptom manifestation; these habits were not considered in our study, which aimed to assess gas station attendants in a broader perspective.

Finally, the study performed by Grendele & Teixeira^19^ shows that the predominant education level among gas station attendants in their sample was incomplete upper secondary education, and no statistical significance was found for the association between schooling and use of PPE since professionals knew which pieces of PPE should be worn in different situations of exposure due to previous training. In our study, no correlation was observed between education level and use of PPE, nor between training and use of PPE. We noticed not only that most gas station attendants had upper secondary education, but also that there were flaws in the training provided by employers; this shows how training is more relevant to the use of PPE by workers than their education level. In addition, we can also infer that flaws may be present in the public education of the studied population, since a higher education level does not correlate with a higher use of PPE, regardless of training.”

### LIMITATIONS

This work was a challenging endeavor due to the difficulty in reaching gas station attendants and their defensive behavior considering the possibility of retaliation by managers and gas station owners. However, even though our sample size was relatively small when compared with the number of gas stations and attendants in the Sorocaba region job market, the researchers believe in the relevance of this theme and of the obtained information. This work is unprecedented in the city of Sorocaba and will certainly contribute to the body of evidence available in the scientific literature on the risk perception by Brazilian gas station attendants regarding their exposure to toxic substances.

## CONCLUSIONS

The final results obtained in this study demonstrated indications of non-compliance by gas station attendants in the Sorocaba region as to the use of PPE at the workplace. Even though employers provide training sessions, these are not always in accordance with the established standards, and a considerable number of employers does not provide any training. The results indicated a possible association between training and use of PPE, where trained gas station attendants are more prone to wearing PPE. The data also demonstrated that the reported symptoms are difficult to be associated with exposure to toxic substances at the workplace, and only a higher number of individuals reporting myalgia was easily correlated with benzene exposure. Altogether, the data presented here show that higher support by the employers and higher awareness by gas station attendants are required in the Sorocaba region when it comes to risks associated with the exposure to toxic substances at the workplace.
